# A new use of technology to solve an old problem: Estimating the population size of a burrow nesting seabird

**DOI:** 10.1371/journal.pone.0202094

**Published:** 2018-09-14

**Authors:** Yuri V. Albores-Barajas, Cecilia Soldatini, Alejandro Ramos-Rodríguez, Javier E. Alcala-Santoyo, Roberto Carmona, Giacomo Dell’Omo

**Affiliations:** 1 Universidad Autónoma de Baja California Sur. La Paz, B.C.S., Mexico; 2 *Berta maris*, La Goleta 330, La Paz, B.C.S., Mexico; 3 Centro de Investigación Científica y de Educación Superior de Ensenada–Unidad La Paz, Miraflores 334, La Paz, B.C.S. Mexico; 4 *Ornis italica*, Piazza Crati 15, Rome, Italy; Phillip Island Nature Parks, AUSTRALIA

## Abstract

Estimating the population of burrow-nesting seabirds is a challenging task, as human presence in the colony creates disturbances and can damage burrows and occupants. Here, we present a novel method using aerial photographs taken with Unmanned Aerial Vehicles (UAVs) to estimate the population size of a burrow-nesting seabird, the Black-vented Shearwater (*Puffinus opisthomelas*), on Natividad Island, Mexico. Our results provide a census of burrows in the colony, with very low detection error (5.6%). This is greater accuracy compared to other methods based on extrapolating results from sample plots to total colony area. We then combined this burrow census with ground truth data on occupancy to estimate population size. We obtained a population estimate of 37,858 and 46,322 breeding pairs for 2016 and 2017 respectively. The proposed method provides a cost effective and repeatable approach for monitoring numbers of burrows occupied in a colony, thereby enabling easier and faster estimates of population trends. We suggest this method can be valid for other burrow-nesting species in habitats without dense vegetation cover.

## Introduction

Seabirds are among the most threatened of bird taxa, with 42% of 365 species listed as threatened on the IUCN Red List [1]. Accurate population sizes and trends are challenging given they often breed in remote sites, are long-lived life-history and have vast at-sea distributions (2,3,4). For burrowing petrels, estimating colony sizes is even more difficult than for surface-nesting seabirds because it requires accurate identification of usually cryptic burrows and nocturnal behavior, where nest occupancy requires ground truth [2, 3]. Nonetheless, robust colony and population estimates are critical for understanding trends and informing conservation management of the status of burrowing seabird populations. Improving the accuracy and repeatability of burrow counting methods, plus lowering the cost and likelihood of disturbance to colonies, remains important to improve population size estimates and understanding population trends.

Several different methods have been used to determine population sizes of burrowing seabirds [4–6]. For burrowing petrels, one of the more common methods is to measure the perimeter of the colony and then extrapolate burrow densities from a sample of plot within the colony [7, 8]. Basic extrapolation methods have been improved by predictive habitat modeling [9, 10], reducing bias due to habitat characteristics, and accuracy of occupancy estimates improved through the use of burrow scopes [11]. Methods based on extrapolation also produce a measure of variance for colony size estimates. Where variance is high, this leads to high uncertainty in estimates, and lower power to detect trends over time, thereby reducing the value of these population estimates for conservation management [12].

In recent years, various field methodologies, technologies, and mathematical models have evolved, allowing researchers to detect, with increased accuracy, variation and changes in population size or demographic parameters, including distance sampling combined with burrow-scoping [13], thermal cameras, and CO^2^ detectors [12], and acoustic monitoring to estimate population size [4]. In recent years, the use of unmanned aerial vehicles (UAVs) or drones, has exponentially increased and are now regularly used for wildlife monitoring, with reduced disturbance of sites and enhanced precision when compared to more conventional methods [14, 15] for counting surface-nesting seabirds [16] and waterfowl [17]. Advantages of UAVs include archiving of images for future comparison and analysis, often less time in the field once the method is established, and less time in the colony itself, thereby reducing costs and disturbance to wildlife [18, 19]. However, to our knowledge, no attempt has been made to count burrows from aerial photography and census-burrowing seabirds.

We used a UAV-based method to estimate the population size of burrow-nesting seabird, the Black-vented Shearwater (*Puffinus opisthomelas*), on Natividad Island, Mexico. The UAV collected imagery, allowing a complete count of burrows in the colony, which we supplemented with on-the-ground estimates of burrow occupancy to determine colony population size. We compared the results of our UAV approach and other population estimates methods to highlight the applicability of using aerial-based methods, and potential application to other species.

## Methods and background

### Ethics statement

All applicable national and institutional guidelines for care and use of animals were followed. Fieldwork was carried out under permits SGPA/DGVS/00321/16 issued January 2016 and extension SGPA/DGVS/003843/16, issued in April 2016 and renewal SGPA/DGVS/00404/17 issued in January 2017.

### Study species and study site

**Fig 1:**
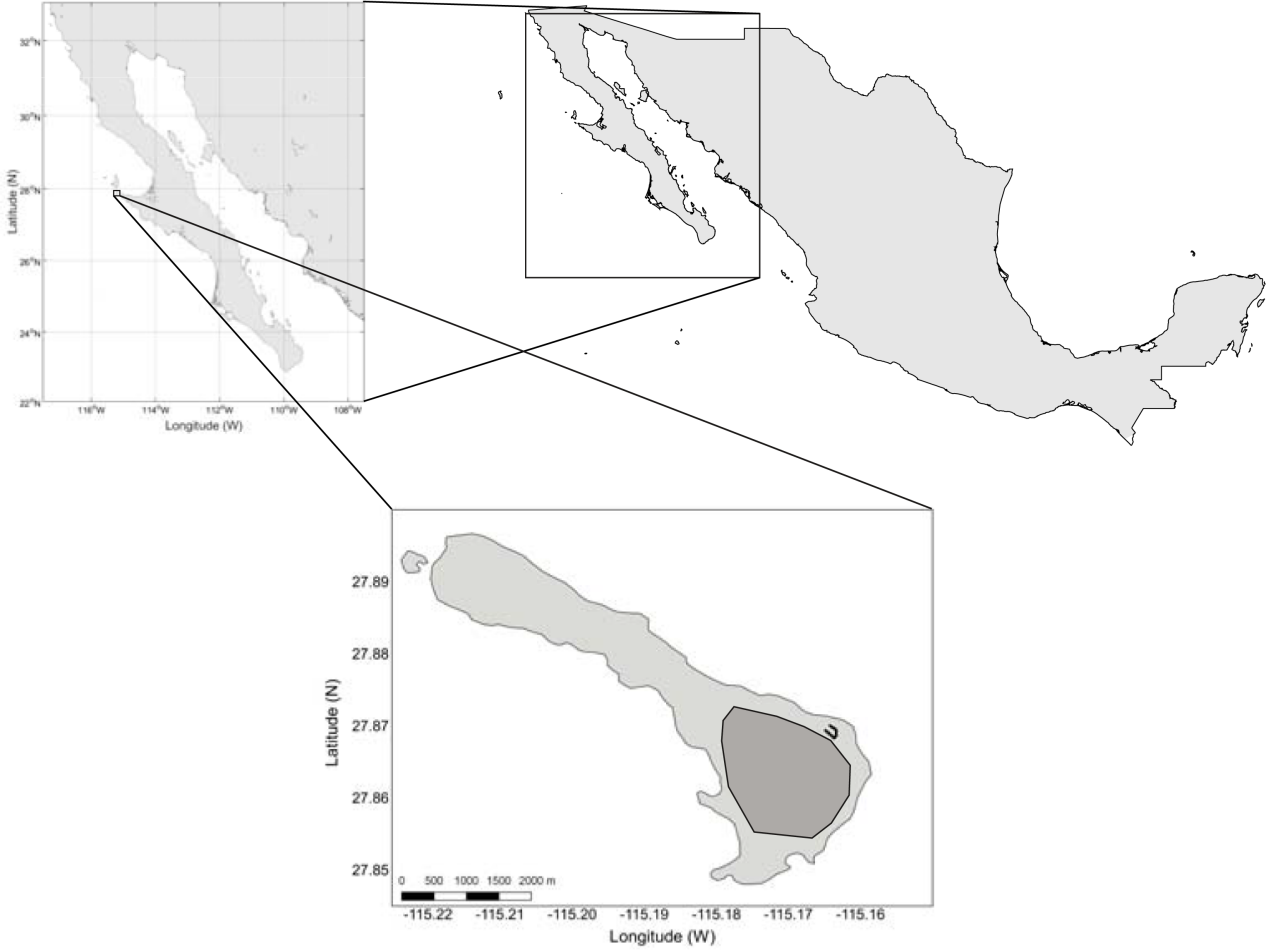
Location of Natividad Island, northwestern Mexico. The Black-vented Shearwater colony on the island is in dark grey.

Natividad Island (27°51′10″N, 115°10′22″W; Fig 1) is five km north of Punta Eugenia in the State of Baja California Sur, Mexico. The island is 8.65 km^2^, arid, with little vegetation [5]. Previous surveys estimated that the island supports 75,000 breeding pairs of Black-vented Shearwater, or 95% of the world’s population [7]. The Black-vented Shearwater is currently listed as Near-threatened on the IUCN Red List [20] and was down-listed from Vulnerable after conservation actions to remove critical threats, including eradication of feral cats between 1997 and 2001 [21]. No population estimates have been conducted since removing feral cats in 2001 [22]. The species also lacks a Conservation Action Plan and long-term monitoring program [1].

Black-vented Shearwaters are present on Natividad Island 10 months of the year, arriving in December and departing in September [23]. They nest in areas without tree cover and dig burrows in sandy soil that are usually deeper than one metre, with nesting chambers usually located 1–2 m below the surface. A small percentage of birds also nest in rocky crevices [23].

In 1998–99, the shearwater colony was estimated to occupy 250 ha on the southeastern part of the island [7]. The area of the colony was estimated, using a handheld GPS (15m accuracy) and mapping the perimeter on foot [7].

### Nest counting by aerial survey

In 2016, we visited Natividad Island three times to update population estimates: 7–14 February, 19 April to 12 May, and 9 June to 3 July. We also visited the island on 11–14 July 2017 to determine the effect of UAVs on ground nesting seabirds. We used a DJI Phantom 3 Standard UAV (China), which was flown in all four visits. The UAV is 49 x 49 cm, with the props mounted, and approximately 20 cm in height. It is white and, when in flight, it has two green lights and two red/orange lights. The UAV weighs 1216 g. The noise produced by the UAV at 2 m distance is 60 dB [15]. Photographs were stored on a Class 10, 32 Gb miniSD card and downloaded after we have completed each day’s flights.

The camera is the standard camera for the DJI Phantom 3 Standard, with a f/2.8 focal point, exposure time: 1/2,000 (at least), and ISO = 100. Each photo is 4,000 x 3,000 pixels, with a resolution of 72 DPI and 32 bits of color. The camera was supported on a gimbal to avoid vibration effects and was pointed 90 degrees downwards, parallel to flat ground.

The first two field trips were used for calibration of the UAV method. Data collected from the UAV during the June 2016 visit were used for the burrow census. As the number of burrows was expected to remain constant within one breeding season (i.e., no more burrows are dug after the laying has started), we did not compare the variation among the three trips. However, occupancy was monitored during all three visits to account for within-season variability. We selected the highest occupancy figure of the three trips to determine the population size. Non-breeders were considered in the population estimate.

To estimate detection error between aerial surveys and on-ground counts, we visited the colony and counted burrows in 20 circular plots (4.37 m radius, area 60 m^2^) chosen randomly in the colony. Concurrently, we collected aerial photographs of these same plots with the UAV at a height of 30 m, and burrows identified later were counted. Each circular calibration plot was marked on the ground with a flag in the center and a metric strip marking the diameter to aid comparison between methods. Comparisons were then made between ground and aerial burrow counts to determine the detection error. Two different persons counted either the burrows from the aerial photograph or the plots on the ground, without exchanging information.

**Fig 2:**
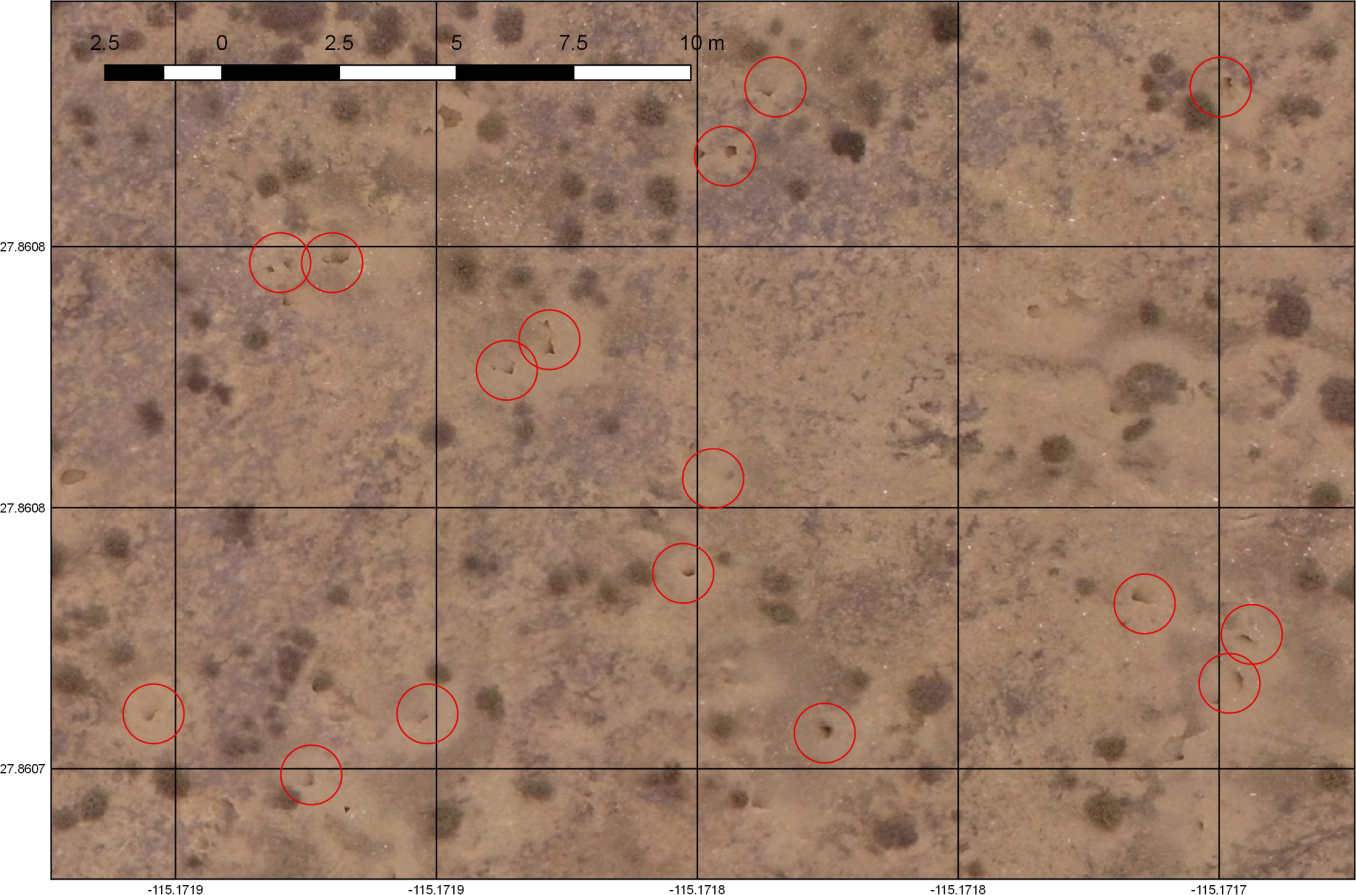
Sample image of the images taken by the drone. This photo was taken at 47.6 m. Red circles indicate some of the burrows present in the image.

To establish a complete count of burrows in the colony, aerial photographs of the entire colony were taken between 25 and 60 m above ground level. Altitude variation was largely an artifact of navigating in the hilly terrain. Given the high resolution of the photographs (average post-processing–see below 27,500 x 27,500 pixels, 96 DPI, and 32 bits of color), this height difference did not appear to affect detection probability, as we were able to zoom in considerably before image resolution limited burrow detection. The center point of each photograph was approximately 10–15 m apart, with each photograph overlapped at least 25%. The UAV was flown at a speed of ≈2 m s^–1^ and a photograph was taken every five seconds. Each photograph contained metadata (30 variables, including GPS location, height, precision estimates, time, date, and photo settings) that were used by the imaging software to locate and arrange all photos. Photographs were taken between 11 AM and 3 PM local time to reduce shadows, which could be mistaken for burrows (Fig 2). During the June–July visit, we used the app “Litchi” (https://flylitchi.com/) to establish the flight path and avoid excessive overlap and large areas without burrows on the edge of the colony. Each flight was programmed to last for 13 minutes and fly a total distance of 3100 m on parallel paths of 300 m long and 20 m apart; each flight covered between 9 and 12 hectares. Weather permitting, the UAV was flown daily. When wind was over 40 km/h, we did not fly the UAV.

We used the photogrammetry software, Agisoft Photoscan Professional [24], to generate orthomosaics from the original photos [25, 26]. The resulting photographs were exported as tagged image file format (tiff) files and were added to QGIS software QGIS Development Team [27]. In QGIS, we added an 11.16 x 11.16 m grid (124.6 m^2^) to use as a reference point when counting the burrows. The grid size is the minimum set by QGIS. We found the optimum scale before image resolution limited burrow detection to be 1:40. Subsequently, all burrows were counted and marked on QGIS. We created a layer on QGIS and marked each burrow with a point over the burrow entrance. Two people carried out the counting independently over a period of 20 working days (8 hr per day). When in doubt, the counters consulted each other to reach a consensus on whether it was a burrow or not. To estimate burrow density, we divided the number of burrows per 124.6 m^2^ grid square.

### Occupancy estimate

Burrow occupancy was estimated using ground-based methods. We used 25 quadrants of 20 m x 20 m randomly selected. Each corner was marked with wooden stakes and locations recorded with GPS. We separated the quadrats in three categories, based on burrow density—low, medium, and high, following Keitt et al. [7] for comparisons. High density areas had ≥ 0.15 burrows per m^2^ (>18 burrows per 124.6 m^2^ quadrat), medium density areas had between 0.10 and 0.15 burrows per m^2^ (between 12 and 18 burrows per quadrat), and low density had <0.10 burrows per m^2^ (<12 burrows per quadrat). Burrows were monitored in April, May, and June 2016, and June 2017, and occupancy was estimated using toothpicks placed at the entrance of the burrows. We monitored 1,342 burrows with toothpicks. The toothpicks were deployed late in the evening, when ravens (*Corvus corax*) and gulls (*Larus occidentalis*) were known to reduce or stop their activity; therefore, they would not interfere with toothpicks. The toothpicks were recovered early in the morning, before ravens and gulls became active. If the toothpick was down, we assumed the burrow to be occupied [7, 28]. The toothpicks were deployed for only one night per field trip. We deployed the toothpicks three times per quadrat in 2016 and once per quadrat in 2017.

Burrow scopes were used to detect whether there were signs of reproduction, “real occupancy”, and to discriminate from nests visited randomly or used by non-breeders, “apparent occupancy”. This method was used to calibrate our occupancy estimates, using the toothpick method. Twenty burrows were inspected inside four of the 20 x 20 m quadrats, for a total of 80 burrows. We used a burrow scope with a SHARP CMOS sensor, 6 white LEDs, 640 x 480 resolution and a 7 mm lens. The burrow scope was connected to a smart phone and photographs taken were stored on the internal memory of the phone.

We could not choose our sample plots randomly because the burrow scope limited our selection to burrows that had shorter and straighter access tunnels (<1 m) dug in solid soil. The burrows were repeatedly inspected in May and June to record activity histories for each nest. We had a similar number of burrows in each of the burrow density groups—low (27), medium (27), and high (26).

We used multi-event capture-recapture models to estimate the proportion of breeders and non-breeders and the probability of detecting evidence of reproduction. For species that have asynchronous laying, such as the Black-vented Shearwater, capture-mark-recapture (CMR) was used to estimate the breeding population size in a given year [29]. Occupancy models were applied to the subset of 80 burrows of data, using E-SURGE, a general software for multi-event capture-recapture models [30, 31]. Nests were sampled on discrete occasions, and they may be occupied or empty. The detection of a nest as occupied has an intrinsic uncertainty on the status of the occupant, which can be a breeder or a non-breeder (prospector). Consequently, we considered this uncertainty in the assessment of state “breeder”. We then used a model with multiple states and uncertainty to determine the proportion of breeders. We considered four states: unoccupied, occupied with no evidence of reproduction, occupied with evidence of reproduction (egg or chick), plus the default state death (necessary for processing data in E-SURGE). There are three categories of events: species undetected (0), detected alone (1), or detected with a chick (2). The non-breeder state represented the model uncertainty [30]. The proportion of non-breeders obtained was subsequently used for estimating the total population breeding in 2016. To estimate the total number of chicks, we applied the probability of finding a young in an occupied nest to the total number of occupied nests obtained with the toothpick method.

### Disturbance and costs

The use of UAVs over a colony can cause stress and evasion behavior by birds [15, 32] depending on the speed, distance, and noise of the UAVs. On Isla Natividad, the Western Gull (*Larus occidentalis*) nest near the Black-vented Shearwater colony and would be disturbed by the UAV. Common Ravens (*Corvus corax*) and Osprey (*Pandion haliaetus*) also fly over the colony during the day. Ravens frequently land in the burrow areas and walk among burrow entrances to prey upon individuals close to the entrance. The gull colony is dispersed throughout the island, with no preferred nesting areas. The population is estimated of over 15,000 individuals (unpubl. data). To determine the effect of the UAV on Western Gulls, we flew the UAV in three randomly selected areas directly over the gull colony in July 2017. We counted the initial number of gulls present in the area and recorded the behavior of the gulls when the UAV flew at elevations of 60 m, 50 m, 40 m and 30 m in a 200 m line transect, with flight time between 100 and 130 seconds. We recorded the time when the first individuals were flushed, the time to return to the nest, the number of reacting birds, and the kind of reaction: alarm (when the individual stopped doing its previous activity) and/or mobbing behavior. All flights were done between 1100 and 1500 hours, the same time the UAV was flown to collect images for burrow counts. During the disturbance study, there was one UAV operator and one independent observer of the gull behavior. The observer was no closer than 30 m from the nearest nest, and gull behavior was not noticeably influenced by the observer. We concentrated on behavioral and not on physiological responses [33].

To make a qualitative comparison between the cost of the UAV method and a traditional population assessment, we budgeted a survey on Isla Natividad from our headquarters in La Paz, Mexico, in days or number of pieces of equipment, including traveling, lodging, days/person, and field material costs. We estimated the cost of a survey, using on-ground only methods and assuming three survey trips to the island, which included mapping 181 sample plots of 60 m2 (the same number used for previous population estimates), counting all burrows within each plot, and measuring burrow occupancy in half of these, plus measuring the perimeter of the island.

## Results

### Burrow counts by aerial survey

**Fig 3:**
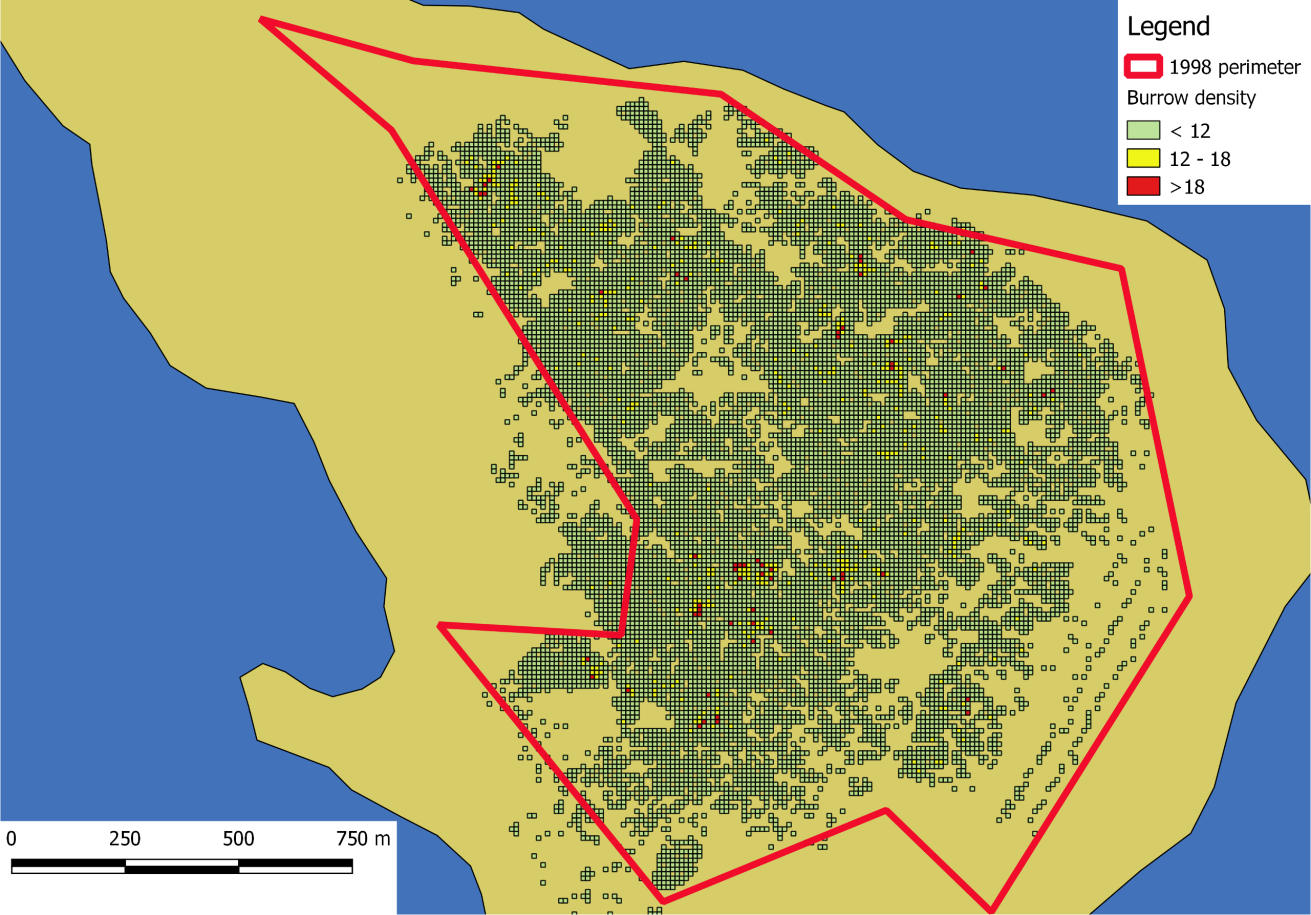
Map of the black-vented shearwater colony on Isla Natividad. The red line is roughly the perimeter of the colony recorded in 1998. Line is for comparison purposes only. The colored grid are the locations of 11x11 m squares with at least one burrow. The shoreline of the island was obtained from CONANP (http://sig.conanp.gob.mx/website/pagsig/info_shape.htm).

**Fig 4:**
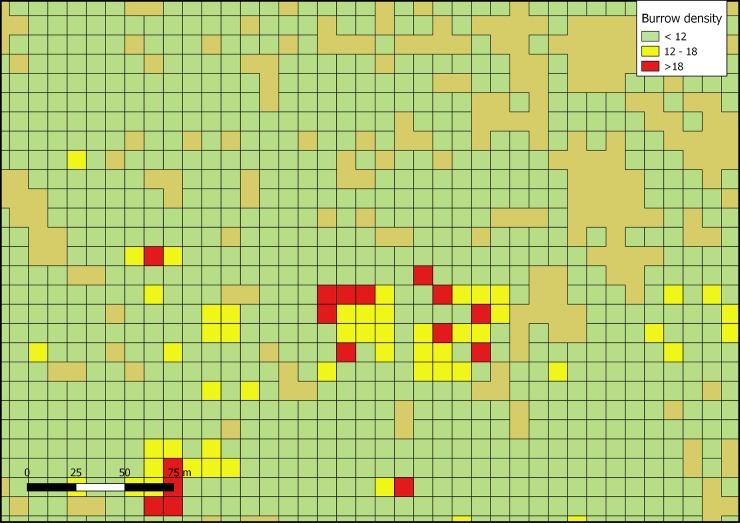
A close up of the density grid obtained from the aerial photographs and projecting them on a 11x11m grid. Many of the areas without burrows are rocky soils, without the possibility to host burrows. These small gaps in the middle (tan cells) might have led to the overestimation in 1998–99.

We flew two to six flights each day with the UAV on most days, depending on wind speed. We covered a total of 300 hectares with aerial photographs, resulting in more than 40,000 photographs. These photographs produced 30 overlapping orthomosaic images extending approximately 150 m beyond the edge of the colony. Within these orthomosaics, the entire colony was represented by 11,963 quadrats of 124.6 m^2^. From the quadrats with at least one burrow, we estimated the area occupied by the colony to be 148 ha (Figs 3 and 4), with 11,315 quadrats having low density (<0.10 burrows/m^2^), 565 having medium density (between 0.10 and 0.15 burrows/m^2^), and 83 having high density (>0.15 burrows/m^2^).

We estimated detection error of the aerial counts of –5.6%, meaning the aerial counts missed 5.6 burrows for every 100, after comparing aerial and ground-based counts within the 60 m^2^ circular calibration plots. The corrected total from the aerial photographs was 56,395 burrows. The area within the colony perimeter was 261 ha, of which 148 ha contained burrows, an increase of 10 ha from the previous estimate that used the perimeter method to estimate colony size [7].

From the grid superimposed on the aerial photographs (11.16 x 11.16 m), we estimated that burrow densities vary from an average of 0.07 (±0.016 SD, n = 9 quadrats) burrows m^–2^ in low density clusters, 0.12 (±0.016 SD, n = 9 quadrats) burrows m^–2^ in medium density clusters, and 0.18 (±0.020 SD, n = 7 quadrats) burrows m^–2^ in high density clusters (Table 1).

**Table 1:** Comparison of conventional, ground-based surveys (1997/98) [7] and a UAV-based aerial photography survey (this study) of Black-vented Shearwaters on Isla Natividad.

	1997/98			2016			
	Low	High	TOTAL	low	medium	high	TOTAL
**mean burrows / sq m ± SD**	0.031 **±** 0.04	0.08 ± 0.07		0.07 ± 0.01	0.12 ± 0.01	0.18 ± 0.02	
**Coefficient of Variation**	127%	84%		23%	13%	11%	
**Area (ha)**	191	65	256	140	7	1	148
**Burrows per density**	60,303	54,152	114,455	46,135	8,407	1,853	56,395

### Occupancy estimate

We compared our toothpick method for estimating occupancy against burrow scope results of true occupancy to determine error in the toothpick method for detecting actual occupancy. The toothpick method resulted in an overestimate of 22% in May (when there were only eggs in the nests) and an underestimate of 16% in June when eggs or chicks were in the nests. Based on burrow depths and soil characteristics, only a small portion of nests could be inspected with the burrow scope, potentially biasing our occupancy estimates. Consequently, for occupancy, we used only the toothpick method.

The average occupancy, based on the toothpick method and calibrated with the burrow scope, was variable in low density zones with 65% (±26% SD) of burrows occupied, and less variable in medium and high-density zones, with 78% (±7% SD) and 77% (±10% SD) of burrows occupied, respectively. In June 2016, following an apparent widespread breeding failure, we observed a decrease of occupancy, compared to earlier in the year, resulting in estimates of: 37% (±16% SD) in low-density clusters, 56% (±17% SD) in medium density clusters, and 37% (±12% SD) in high-density clusters. The occupancy rate in 2016 is similar to the 1997 estimates, but much higher than in 1998 [5], see Table 2). Estimates for 2017 were higher than 1997, 1998 and 2016.

**Table 2:** Comparison of the conventional survey method used in 1997/98 and the UAV method used in 2016.

		Colony size, burrow number, and density estimate, as determined by two methods	
	Source	1997/98 [7]	2016 (this study)
Colony size	method	walking perimeter	aerial photograph of entire colony area
Colony area	area (km2)	2.56	1.48 (walking perimeter 2.61)
Burrow count /estimate	method	181 plots of 4.37m radius (60 m2), to determine no. burrows per sq km in LBD areas (n = 88), and HBD (93). Mean density then extrapolated to determine total burrow count	Counted all burrows in photographs. Split total colony area into 11,963 x 124m2 quadrats based on photos and counts of >0.15 b/m2 = high burrow density (n = 83), >0.10 & <0.15 = medium burrow density (n = 565), and <0.10 = low burrow density (n = 11315). Excluded plots with zero density, which essentially is a 4^th^ category.
	area surveyed (ha)	1.086	148
	% colony surveyed	0.424%	100%
	No. burrows	114,455	56,395
	Lower bound estimate	86,935	no error estimate because number is total count of colony
Occupancy	Method	Using same 181 plots described above, burrow scope of all burrows	Surveying 25 x 400 m2 quadrats (9 in LBD, 9 in MBD, 6 in HBD), using tooth-picking determine status and burrowscope to calibrate.
	Occupancy (year)	66.9% (1997), 19.6% (1998)	67.1% (2016), 82% (2017)
Population Size Estimate (breeding pairs)		76,570 (1997), 22,433 (1998)	37,858 (2016), 44,235 (2017)

When applying multi-state occupancy models [30] during the reproductive period, we assumed that nests were most probably occupied by breeders (87.5%), but in the population, there is a small portion of non-breeders visiting the nests (12.5%) in both years. From the same models, we estimated the probability of detecting a breeder as 40% and the probability of detecting a non-breeder, inspecting a nest during the early breeding phase at 7%.

### Population estimates

The Natividad Black-vented Shearwater population was 37,858 (±8,510 SD) breeding pairs in 2016, and 46,322 (±4931 SD) in 2017. When adding non-breeders, the estimated number of individuals visiting the colony was 84,802 in 2016 and 103,761 in 2017.

### Impact of the UAV on other birds

Flights were conducted after Western Gulls chicks had already hatched in three sample areas (a, b, c) of the colony with 90, 75, and 98 nesting adult individuals, respectively. We saw little evidence of behavioral reaction to the UAV flying at 50 m above ground and no evidence of reaction at 60 m. Behavioral reactions in response to lower altitude flights consisted of alarm calls and increased vigilance, observed in ≈1 to 5 of 263 birds. Between 0 and 2 birds flushed in response (Table 3). Based on anecdotal observations, we saw no response from ravens or osprey to the UAV.

**Table 3:** Results of the disturbance experiment of three UAV flights in the post-hatching period of the Western Gull. Flights were done at four altitudes. The total number of individuals in the areas were counted before the flights started (N = 263). The number of gulls remained constant during the trials, with no individuals coming into or leaving the colony.

UAV flight height	% of individuals showing reaction	
	Reacted, but stayed on the nest	Flushed
30 m	2±5.5	0.1±1.2
40 m	2±4.1	0.6±1.2
50 m	0.3±0.6	0
60 m	0	0

### Cost comparison

We estimated that our UAV-based survey of burrowing seabirds represented a 68% savings in man-hours over traditional ground counts, even when accounting for the additional time required for UAV image processing (double the research assistant time; Table 4). For example, we estimated that a team of five people would require 17 days to cover the 181 circular survey plots (1.09 ha). The UAV covered 12–13 hectares in 13 minutes. We estimated that a traditional ground survey would require a team to spend an additional 35 days in the field. While the UAV survey was more expensive (cost of the UAV), future additional UAV-based surveys would represent an even greater savings, since no purchase is necessary.

**Table 4:** Comparison of survey costs, in terms of time spent traveling to or on the island for personnel, lodging, meals, transportation, and materials, between the two survey methods: the traditional nest counting for sample units and UAV. The traditional method estimates are for a highly accuracy survey, comparable to the UAV method. The duration of the working days was eight hours each day.

Category	Conventional survey			UAV survey			difference (conventional-UAV)
**Personnel**	units	Days or number	total	units	Days or number	total	
senior researcher (field work)	1	50	50	1	15	15	35
senior researcher (data analysis)	1	5	5	1	5	5	0
research assistant (field work)	4	50	200	1	15	15	185
research assistant (data analysis)	1	30	30	2	30	60	-30
**Lodging**							
on island	5	50	250	2	15	30	220
traveling to the island	15	2	30	2	2	4	26
**Meals**							
on island	5	50	250	2	15	30	220
traveling to the island	15	2	30	2	2	4	26
**Transportation**							
Gas tanks	3	3	9	1	3	3	6
**Field material**							
Burrow scopes	1	5	5	1	2	2	3
UAV Phantom 3 Standard	0			1	1	1	1
software Litchi licence	0			1	1	1	1
GPS	3	1	3	1	1	1	2
metric tapes + sticks and signs	3	3	3	1	1	1	2
desktop computer	1	1	1	1	1	1	0

## Discussion

Accurate population estimates and the ability to easily track trends in populations are key to effective management of threatened species. Here we describe the first use of UAVs to capture images to facilitate an accurate and repeatable count of seabird burrows on a breeding colony, which are difficult to monitor. The advantages of this approach include: (a) a complete burrow census and thus greater power in detecting changes over time, (b) archivable, as images are available for future reference, (c) data can be collected without damaging burrows, i.e., only limited walking is needed in the colony, (d) data are reproducible and relatively inexpensive to collect.

### Nest count by aerial survey

Our nest census showed 56,395 burrows, which combined with an occupancy estimate, provided an estimate of 84,802 individuals in 2016. These results are not directly comparable to the Keitt et al. [7] estimate of 1997/1998 because of they used different methods. The main difference was that the aerial approach allowed us to exclude areas of zero burrow density. Such exclusion was not possible in the ground-based method of Keitt et al. [7]. Multiplying the three density estimates in our study and the area of the colony, the estimate of 114,000 to 108,000 burrows is similar to Keitt et al. [7] (Table 2).

It is notable that the colony area, when using the method of including all the area within the bounds of the colony (Fig 3), is similar in our study (Table 2) and the Keitt et al. [7] study. The 100 ha difference in area reported in our study, corresponding in a reduction in area of 42.8% from that of Keitt et al. [7], is primarily due to the exclusion of survey plots with zero burrows within the bounds of the colony.

Unmanned aerial vehicles have been used to estimate population of ground-nesting seabirds [16]. For Lesser Frigatebirds (*Fregata minor*), Crested Terns (*Thalasseus bergii*), and Royal Penguins (*Eudyptes schlegeli*), the estimation error was ±10% [16]. However, the methods have been improved and now have a much lower detection error [34], comparable to the 5.6% detection error we obtained in our study.

### Occupancy estimate

We found that the toothpick method underestimated Black-vented Shearwater burrow occupancy, compared to actual occupancy, as determined with the burrow scope. This is likely because of the Black-vented Shearwater nest- attending behaviour, where parents leave the nest unattended for two or more days or remain inside for several days. Toothpicks would not be knocked down on those survey days, and nests would be classified as unoccupied. We chose to use the toothpick data in the population estimate because it provided a larger and more variable sample of nests, including many types of soil, vegetation cover, and different areas within the colony. Burrow scope inspection was limited only to suitable nests in terms of soil type and length of the nest chamber.

We included a non-breeder state in our models [30] to account more accurately for the proportion of the population that skip a breeding year, which may be an artifact of individual lower body condition or unfavorable environmental conditions, such as a post-El Niño year [35, 36]. From the E-Surge modelling exercise, we estimated that 12% of the population in the colony were non-breeders, a percentage we expect would be lower in years with favorable environmental conditions that increase the likelihood of reproduction. We also estimated 44% of the active burrows were occupied by a chick in June 2016, giving us an estimate of the species productivity under post-El Niño conditions. June is generally late in the normal breeding schedule [23]. A similar delay was described by Keitt and colleagues after the El Niño event of 1998 [7].

Occupancy rates can change dramatically under El Niño conditions, compared to other years [7]. Thus, estimating the bird population from one year of survey during an El Nino is challenging. The numbers from 1997, and 2016 should be considered a minimum population estimate as the surveys were done during unfavourable conditions. We suggest it is more useful to include a measure of occupancy with burrow counts, rather than only counting physical burrows, because this will produce a more biologically relevant value to compare over time. Furthermore, there is always a proportion of burrows that are not occupied and could lead to an overestimation.

### Cost comparison and disturbance effects

The time spent in the field to carry out the UAV survey is much less than the time spent in a traditional survey (Table 4) and will be directly reflected in the funding needed to complete the survey. Also, by spending less time in the colony area and without walking among the burrows, trampling will be much lower. However, the costs of the traditional method can be lower if instead of covering the whole colony area, only randomly selected plots are used but is significantly lower in the accuracy [37].

We suspected that gulls would assess the UAVs as a potential predation risk, making subsequent decisions about fight or flight [38]. We observed little to no change in Western Gull behavior to UAV flights. No individuals abandoned their nesting attempts; the few individuals that flushed from nests returned immediately after the UAV passed.

Similar behaviour has been observed in other surface nesters when approached by a UAV, in some cases passing as close as 4 m from the individual, without causing the bird to flush [15]. While we did not monitor physiological stress responses; such responses can occur, even in the absence of behavioural responses [39], such as increased heart rate and production of stress hormones [32, 40].

The UAV method could also be applied to populations of other burrowing seabird species, especially those that breed in sandy soil free of heavy vegetation, such as the Peruvian Diving-petrel (*Pelecanoides garnotii*) or the African penguin (*Spheniscus demersus*). For colonies where part of the area is obscured by vegetation, a subsample of an accessible part of a colony could be monitored to detect changes. For future research, we suggest determing the best times for taking photographs. We decided to take them between 11 AM and 3 PM to have azimuthal light and reduced shadows. However, this may not be the best approach, as slope or latitude may influence the resulting photographs. To monitor changes in colony population over time, the method we are proposing is less accurate than a census of the whole colony, but it offers a much cheaper option with less likelihood of disturbance.
